# QuickBird image-based estimation of tree stand density using local maxima filtering method: A case study in a Beijing forest

**DOI:** 10.1371/journal.pone.0208256

**Published:** 2018-12-13

**Authors:** Shuhan Wang, Xiaoli Zhang, Mohammed Abdelmanan Hassan, Qi Chen, Chaokui Li, Zhiguang Tang, Yanjun Wang

**Affiliations:** 1 Key Laboratory of Precision Forestry, Forestry College, Beijing Forestry University, Beijing, China; 2 National-Local Joint Engineering Laboratory of Geo-spatial Information Technology, Hunan University of Science and Technology, Xiangtan, Hunan, China; 3 Department of Geography, University of Hawai’i at Manoa, Honolulu, Hawai’i, United States of America; College of Agricultural Sciences, UNITED STATES

## Abstract

The stand density of trees affects stand growth and is useful for estimating other forests structure parameters. We studied tree stand density in Jiufeng National Forest Park in Beijing. The number of spectral local maxima points (NSLMP) calculated within each sample plot was extracted by the spectral maximum filtering method using QuickBird imagery. Regression analysis of NSLMP and the true stand density collected by ground measurements using differential GPS and the total station were used to estimate stand density of the study area. We used NSLMP as an independent variable and the actual stand density as the dependent variable to develop separate statistical models for all stands in the coniferous forest and broadleaf forest. By testing the different combination of Normalized Difference Vegetation Index (NDVI) thresholds and window sizes, the optimal selection was identified. The combination of a 3 × 3 window size and NDVI ≥ 0.3 threshold in coniferous forest produced the best result using near-infrared band (coniferous forest R^2^ = 0.79, RMSE = 12.60). The best combination for broadleaf forest was a 3 × 3 window size and NDVI ≥ 0.1 with R^2^ = 0.44, RMSE = 9.02 using near-infrared band. The combination of window size and NDVI threshold for all unclassified forest was 3 × 3 window size and NDVI ≥ 0.3 with R^2^ = 0.70, RMSE = 11.20 using near-infrared band. A stand density planning map was constructed using the best models applied for different forest types. Different forest types require the use of different combination strategies to best extract the stand density by using the local maximum (LM). The proposed method uses a combination of high spatial resolution imagery and sampling plots strategy to estimate stand density.

## Introduction

Stand density is an important factor that characterizes forest structure. Accurate extraction of forestry density is critical for sustainable forest management. The true stand density can be represented by stem density or tree density or the number of trees per unit area (NT) [1–2]. The stand density used in our study is the number of trees per unit. However, ground-based measurements require substantial labor and resources [3], and they can lead to repeated measurements, which introduce bias and increase variability [4–5]. High spatial resolution remotely sensed imagery is an alternative method for determining stand density because its spatial resolution is sufficient to identify the tops of individual trees [6–8]. Remote sensing tools have been used to estimate stand density by identification of individual trees [9]. Gougeon [10–11] used spectral imagery with a pixel size of 31 cm × 31 cm to identify individual trees in plantations of red pine (*Pinus resinosa*), red spruce (*Picea abies*), white spruce (*Picea glauca*), and Norway spruce (*Picea abies*). This method had a stand density accuracy ranging from 11% underestimation to 5% overestimation. Gougeon also examined a 49-year-old Douglas fir (*Pseudotsuga menziesii*) ranging in density from 650 to 1750 stem / ha and achieved stand density estimates with an average error rate of 37% using 60 cm spectral imagery [12]. Gougeon and Leckie used 30 cm imagery to estimate density in 3- to 10-yr-old jack pine (*Pinus banksiana*) and Scots pine (*Pinus sylvestris*) forests and achieved an average error rate of 21% [13]. McCombs et al. noted that previous studies using remote sensing data compared field-measured stand density to derive stand density without ascertaining whether the identified trees actually existed [14]. A number of studies have focused on species diversity [15–16] and estimation of diameter distribution, tree size distribution, and spatial distribution of trees [17–21]. The general approach to estimate stand density using remote sensing images usually employs spectral values of remote sensing images and stand density to establish a linear or non-linear relationship [22]. However, the correlation coefficients between these dimensional diversity indexes and remote sensing images have not been satisfactory. For example, Ozdemir et al. used ASTER (Advanced Spaceborne Thermal Emission and Reflection Radiometer) multispectral imagery to survey tree dimensional diversity, and the highest correlation coefficient between Gini coefficients from pure UK pine tree stands and texture parameters was 0.69 [23]. A second-order texture image proposed by Pasher and King was used to model forest structure and analyze redundancy using remote sensing data, the coefficient of determination (R^2^) was 0.35 [24]. Multispectral images have been widely used in the modeling and mapping of traditional forest structure parameters, such as stand volume, biomass, tree height, leaf area index (LAI), breast-height basal area (BA) and dimensional diversity indexes [25–26]. The use of spectral signals alone to estimate stand density frequently produces low-accuracy results. Object-based image analysis methods have been evaluated in several reports [27–28]. Additional texture information can improve the accuracy of classification, and methods for selecting appropriate variables from textural features to establish a relationship with vegetation variables of interest is a major goal [29–30]. For example, Chopping made use of full-color images to extract parameters such as stand density, canopy cover and tree forest height [31].

The method of LM filters is simple when using on high spatial resolution remotely sensed imagery without considering the accuracy problem. The tree canopy position detection method using LM currently has some disadvantages when representing the positions of different trees. The aim of the present study was to establish a stand density extraction method using a regression model. The model considered the number of spectral local maxima points (NSLMP) obtained with the LM filtering method by counting the NSLMP within a plot extracted from a high spatial resolution remotely sensed imagery and true stand density obtained from field observations. We explored QuickBird-2 remote sensing data (acronym for QB in the context below) with different bands using different window sizes from LM and NDVI threshold values to improve the estimation accuracy using an optimal window size and NDVI threshold. The optimal strategy was confirmed by stand density estimation models. The unit of stand density is normally given as trees per hectare, i.e. trees / ha. In this study, the image was divided into a 20 m × 20 m grid. Thus, the standard units for stand density in some figures were converted to trees / ha.

## Materials and methods

### Materials

**Fig 1:**
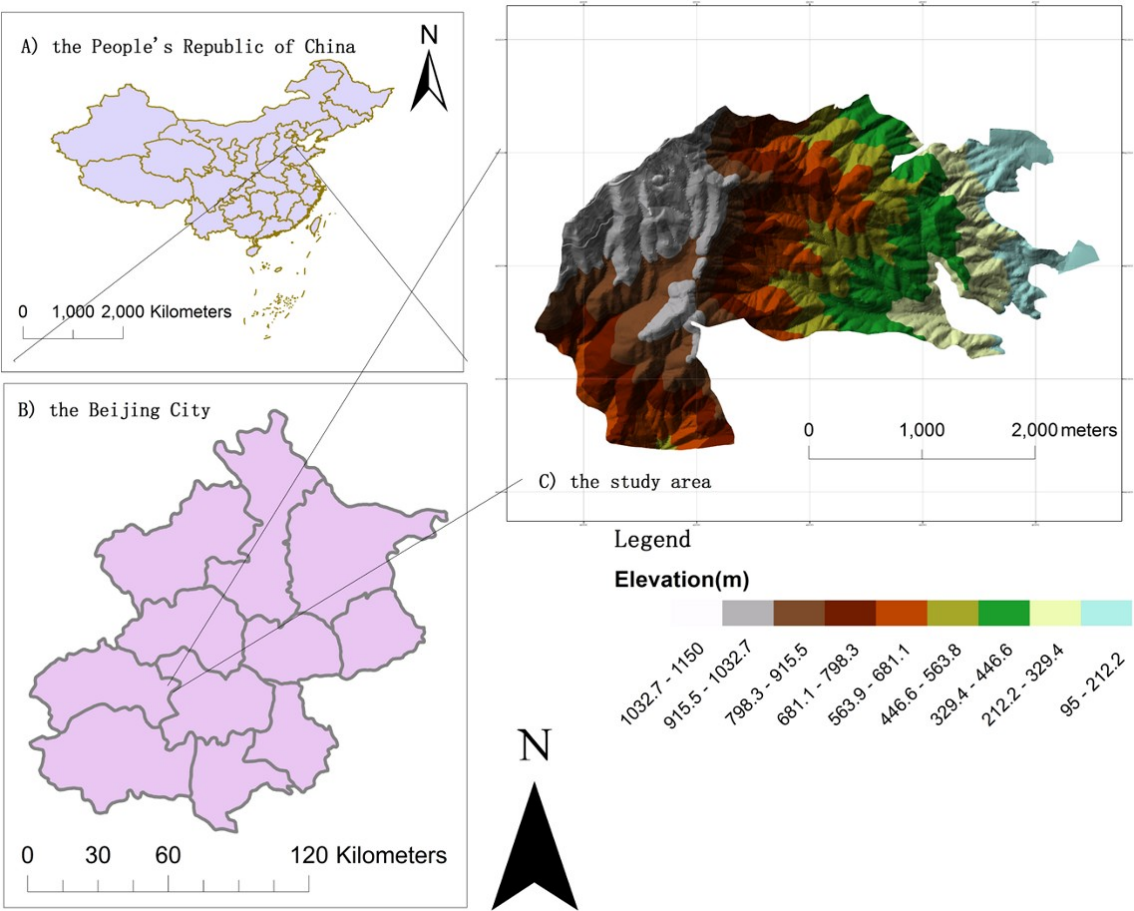
Study area and DEM of Jiufeng National Forest Park, Beijing, China.

The study was conducted in the Jiufeng National Forest Park (39° 54′ N, 116° 28′ E), Haidian District, Beijing, China (Fig 1). Jiufeng National Forest Park is managed by the Forestry Committee of Beijing Forestry University and it is owned by Beijing Foresty University. This site is available for teaching and research use by the university. This study did not involve any endangered or protected species. No specific permits were required for the described field studies. Because this site is an important base of Beijing Forestry University for teaching, scientific research and practice. Teaching and scientific research service are the main tasks of the forest park.

The park covers 811.173 hectares and varies dramatically in topography, with a maximum elevation of 1153 m and minimum elevation of 60 m [32]. Naturally regenerated trees are rare in this forest, and most species, including *Pinus tabulaeformins*, *Platycladus orientalis*, *Robinia pseudoacacia*, *Quercus variabilis*, and *Quercus aliena* were planted in the 1950s and 1960s. *Phellodendron amurense Rupr*. and *Larix gmelinii* are distributed in clumps in the high-elevation region. The average stand density is 675 trees / ha (27 trees / 400 m^2^), the average tree height is 6.6 m, and the average diameter at breast height (DBH) is 11.2 cm. The study area has a sub-humid continental climate with cold dry winters and hot rainy summers. Forest stands in the upland are fragmented with patches of dense shrubs, whereas forests in the lowland are relatively uniform and continuous.

One of QuickBird images, dated on Oct. 24th 2008, was acquired. The sun elevation angle was 37.3°. The sun azimuth was 166.6°. Satellite azimuth was 74.6°. The satellite elevation angle was 67.3°. Another QuickBird image, dated on August 25th 2013, was collected. The sun elevation angle was 53.4. The sun azimuth was 135.7°. The satellite azimuth was 282.8°. The satellite elevation angle was 60.8°. The QuickBird standard product consisted of one panchromatic image (PAN) with a wavelength interval of 450–900 nm and one multispectral image with blue band (wavelength interval 450–520 nm), green band (wavelength interval 520–600 nm), red band (wavelength interval 630–690 nm) and near-infrared band (NIR) (wavelength interval 780–900 nm).

### Data preprocessing

Orthorectification using ephemeris and attitude data measured on the board of a satellite without ground control points (GCP) can only provide an absolute accuracy of about 20 m to 1 km (depending on the satellite). GCP-based orthorectification can obtain more accurate precision than RPC file-based orthorectification [33–34]. Road intersections, houses, and other obvious features were selected as GCPs which were measured by a Handheld differential globe position system (DGPS) in the field work. Nine GCPs were selected for multispectral imagery and 7 GCPs were selected for panchromatic imagery. The digital elevation model (DEM) was derived from a triangulated irregular network (TIN) created by the contour lines extracted from the topographic map shown in Fig 1. The topographical map was surveyed by the Beijing Institute of Surveying and Mapping in October 2004.

### Plot location method

Each tree was located and measured in the field using total station and DGPS. Qualitative information were collected, such as tree species type, tree crown diameter, and DBH. The crown of each tree in the plot was measured from two orthogonal axes using tapes. Plots were collected in July 2011. A total of 73 square plots, each with an area of 400 m^2^ were selected to represent different characteristics of stand density in the study area. They were divided into 26 coniferous plots and 45 broadleaf plots according to the percentage of the coniferous or broadleaf tree component in each plot. The average stand density was 675 trees / ha (27 trees / 400 m^2^). The minimum stand density was 100 trees / ha (4 trees / 400 m^2^). The maximum stand density was 2750 trees / ha (110 trees / 400 m^2^) and the stand deviation (SD) of stand density was 500 trees / ha (20 trees / 400 m^2^).

The average crown diameter of the coniferous plot was 2.68 m (North to South) and 2.95 m (East to West). The minimum crown size was 1.38 m. The maximum crown was 6.61 m. Average crown size of broadleaf plot was 3.63 m (North to South) and 3.79 m (East to West). The SD of the coniferous crown size was 0.96 m. The SD of the broadleaf crown size was 1.52 meters. A handheld differential GPS S740 manufactured by The South Surveying and Mapping Co., LTD in Guangzhou China was used for the field work. The geolocation measurements of single trees were performed with a Pentax R-422N, which can measure distances over 550 m in no prism mode. Thus, precise and quick measurements results were obtained in field surveys using total station surveying. A Beijing 54 coordinate system was used in the study. The coordinate system commonly used in China include Beijing 54, Xian 80 and WGS 84 etc. Beijing 54 is based on Krassovshy ellipsoid and adopts a Gauss-Kruger projection with 117°E central longitude and proportionality factor of 1.0.

### Individual tree identification method

If the grayscale value of a pixel in the image slice is defined as elevation and the image is taken as three dimensions, a tree crown will have a peak point, i.e. LM. The peak point is the LM reflectance within a certain range of elevation, and the value of this point is greater than that of other points. We used LM reflectance to represent spectral reflectance maximum points and to identify the center of individual tree crowns. In other studies, peak extraction methods have been used as an alternative to LM filtering, and this technique has been adopted to detect individual trees [35]. The LM filtering method can be used in different data sources, such as QuickBird panchromatic bands, or Worldview-2 panchromatic bands.

#### NDVI threshold and window size selection

The spectral reflectance peak maximum points extracted by the LM algorithm were not all derived from vegetation points. Points incorrectly located on a road were considered as pseudo crown points. Normalized difference vegetation index (NDVI) filtration removed these pseudo crown points. A threshold NDVI value was set for removing the pseudo points. The tree crowns in each plot were then identified by spatial association statistics. The number of trees in each sample plot was counted by overlapping the sample plot layer with canopy reflectance maxima layers, and the stand density was calculated using a spatial statistics method combining the plot areas. The QuickBird panchromatic band was analyzed by LM filtering first since the filter's window size affects the results. We also tested the effect of varying the window size, using 3 × 3, 5 × 5, 7 × 7, 9 × 9 and 11 × 11 window sizes. The unit of window size here is pixels. The optimal window size was required for identification. The raster layer obtained after filtering was subtracted from the original panchromatic layers, and the points with a value of zero represented the maximum canopy reflectance points. We determined how different NDVI threshold values affected the analysis. We choose five different ranges of NDVI. The first range was NDVI ≥ 0.1. The second range was NDVI ≥ 0.2. The third range was NDVI ≥ 0.3. The fourth range was NDVI ≥ 0.4. The fifth range was NDVI ≥ 0.5. Points under the threshold value were removed to refine the reflectance maximum points layer by referring to the NDVI layer. The spectral reflectance maximum point layers were overlaid on the sample plots, and the number of canopy reflectance maximum points (N') was calculated inside each plot. To identify the optimal combination of NDVI threshold and window size, we used Pearson correlation analysis to evaluate each effect of different NDVI threshold ranges and window sizes. A correlation analysis was then performed between the NSLMP within the plot and the number of trees within the corresponding plot. We took 73 sample plots to evaluate the stand density of all stands; 45 in broadleaf stands and 26 in coniferous stands, and the rest were removed.

#### Statistical analysis

Pearson's correlation coefficient was used to analyze the linear correlation between variables X and Y, which are often two continuous variables. Pearson's correlation coefficient, when applied to a sample, is commonly represented by the letter r. Two-tailed t-tests were used to determine whether the correlation was statistically significance, and p = 0.05 was used as the threshold.

The highest r value indicates the best combination of NDVI threshold and window sizes and the band. After selecting the best NDVI threshold value, optimal window size and band, considering that there is only one explanatory variable, a simple regression with one element was used to build model. NSLMP was treated as the independent variable and the true stand density was treated as dependent variable. A non-linear regression model and linear regression model were both tested.

We used the Shapiro-Wilk test to verify the distribution of the residuals and a regression model was established according to the assumption of a normal distribution of the residuals (predicted value minus observed value). If the p-value was significantly greater than 0.05, then the residuals were distributed normally. The produced models were evaluated for precision using the coefficient of determination (R^2^) and the root mean square error (RMSE). RMSE measures the differences between values predicted by a model and the values actually observed. The unit of RMSE is the same to the unit of stand density. We used the leave-one-out cross-validation approach to calculating cross-validated coefficient of determination (r^2^_cv_) and root mean square error (RMSEcv) to validate the models in predicting the forest stand density. The prediction value of i-th observation was calculated using the regression equation obtained by fitting the model leaving the i-th observation out.

#### Stand density mapping

According to tree species composition in each plot, all plots were divided into three types, pure broadleaf forest, pure coniferous forest and unclassified forest. Broadleaf forest and coniferous forest were considered separately. According to the technical regulations of the National Forest Inventory (NFI) in China, the definition of a pure forest is one in which a single woodland species has more than 65% of total stock volume. The definition of a mixed forest with coniferous and broadleaf trees is one in which no tree species (Group) have more than 65% of the total volume. In our study, we increased the percentage to maintain the consistency of tree species. If the percentage of broadleaf trees exceeded the seventy percent, the plot was grouped as a pure broadleaf plot. And the percentage of coniferous trees exceeded the seventy percent, this plot was grouped as a pure coniferous plot. All of the plots are considered to be unclassified forest. Inventory data in the study area were used as reference data. The study area was divided into different compartments according to the inventory map as reference data. After the estimation models were established, we used Create Fishnet tools in ArcGIS 10.1 to create the 20m × 20m grid. The rectangles were evaluated by using the Spatial Join tool of Analysis tool in ArcGIS 10.1 to count the number of the local maximum in the whole study area. Then, we changed the fishnet vector layer with points count into a raster layer. We used these models to estimate the stand density in the whole area.

## Results

### Image quality

The QuickBird multispectral images at 2.4-m resolution and panchromatic images at 0.6-m resolution and topographical map were geometrically registered into the same coordinate system. The final total correction of the multispectral images was controlled within one pixel at 0.99 RMSE. The RMSE of the panchromatic image correction was 5.86. The sample plot was precisely located on the image after the orthorectification process.

The final total correction of the multispectral images was controlled within one pixel at 0.59 RMSE using the 2008 QuickBird multispectral band as reference imagery. The RMSE of the PAN image correction was 2.59 using the 2008 QuickBird panchromatic imagery as reference imagery.

### NDVI threshold and windows size selection

**Fig 2:**
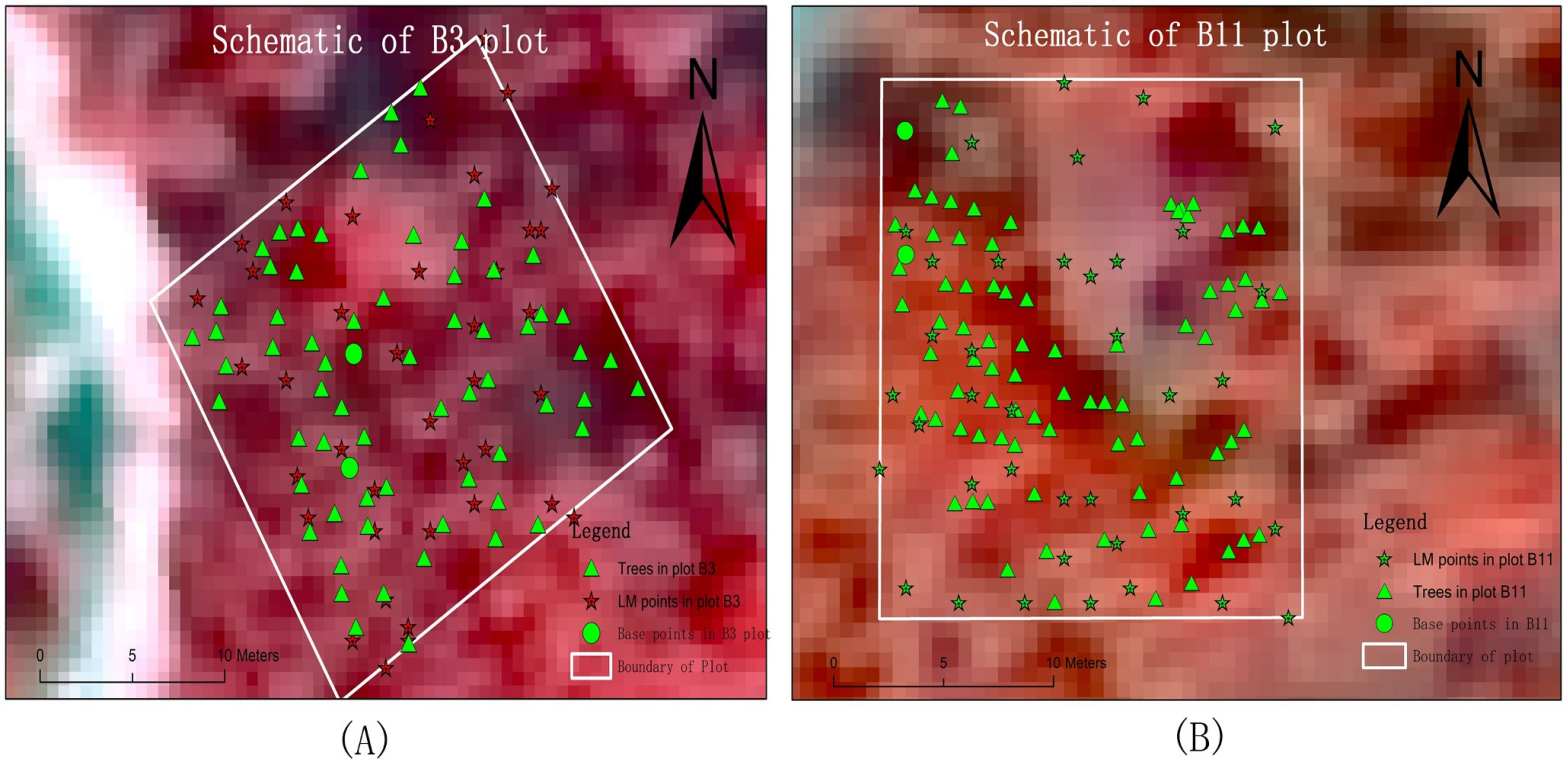
Sample plots geolocation in QuickBird fusion imagery with multispectral and panchromatic band and NDVI threshold is 0.2. (A) Sample plot B3. (B) Sample plot B11.

Two plots were used as samples to evaluate the ability of individual tree identification by LM filtering (Fig 2). Stars represent the estimated crown points from LM filtering and triangles represent the actual tree stem positions in the plots. The spatial resolution of both imageries is 0.6 meter.

According to the results of the Shapiro-Wilk tests, residual distributions, the stand density had a normal distribution. The mean NT was 675 trees / ha (27 trees / 400 m^2^); the standard deviation was 500 trees / ha (20 trees / 400 m^2^); the minimum value was 100 trees / ha (4 trees / 400 m^2^), and the maximum value was 2750 trees / ha (110 trees / 400 m^2^). The 73 sample plots represent most of the structural features of trees in the study area. The Pearson correlation coefficient is suitable for correlation evaluation between variables, and we tested the correlation coefficients between the quantity of spectral canopy maxima extracted from the QuickBird panchromatic band using different window size and the NDVI threshold value and the true NT calculated from the field data. We used the panchromatic and multispectral bands.

**Fig 3:**
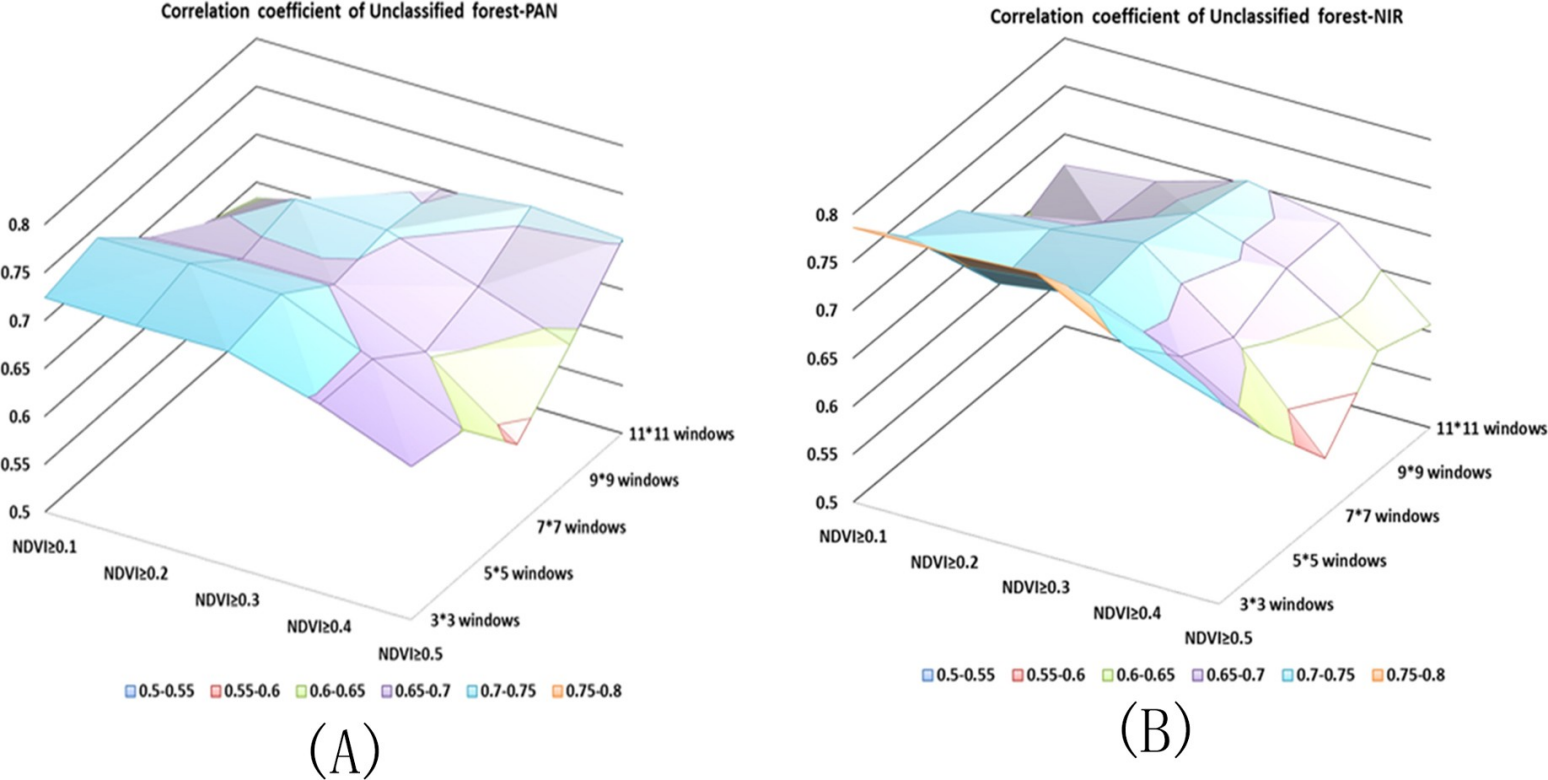
Correlation Coefficients of unclassified stand between stand density based on different window sizes and NDVI threshold and true stand density. (A) Correlation coefficient of the PAN. (B) Correlation coefficient of the NIR.

**Fig 4:**
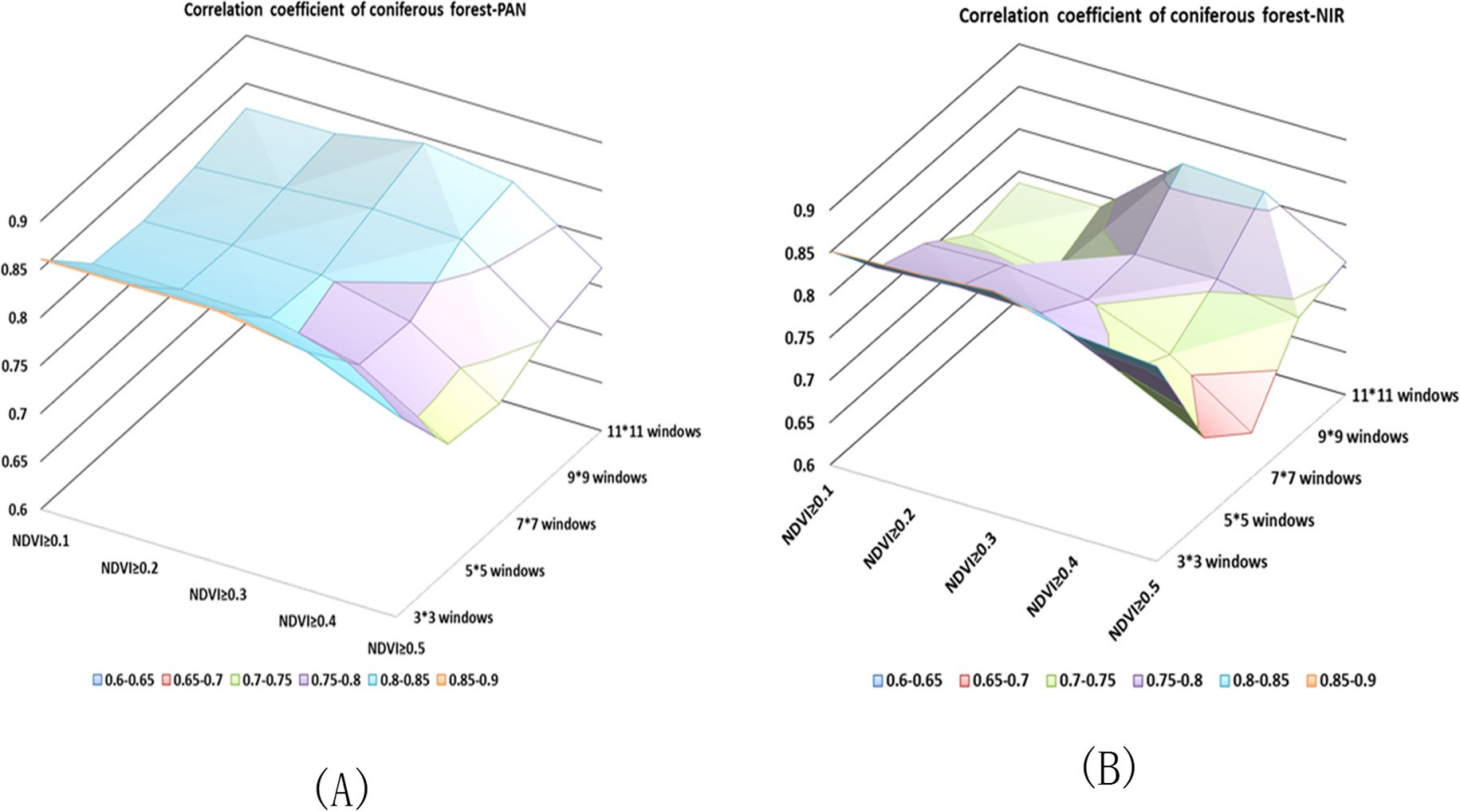
Correlation Coefficients of coniferous stand between stand density based on different window sizes and NDVI threshold and true stand density. (A) Correlation coefficient of the PAN. (B) Correlation coefficient of the NIR.

**Fig 5:**
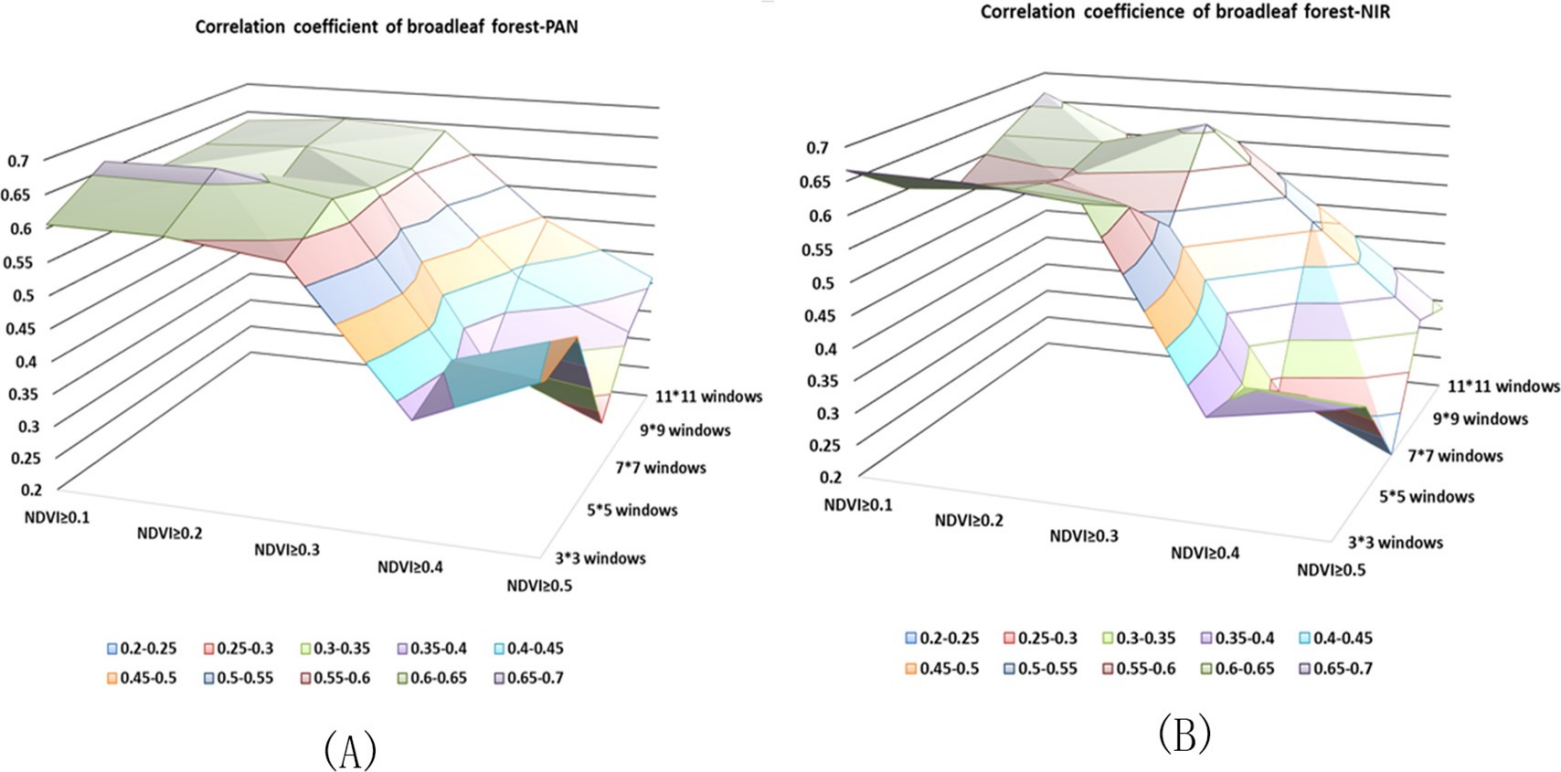
Correlation Coefficients of broadleaf stand between stand density based on different window size and NDVI threshold and true stand density. (A) Correlation coefficient of the PAN. (B) Correlation coefficient of the NIR.

Figs 3–5 illustrate the correlation coefficient of different combinations using the panchromatic band and multispectral band respectively in 2008. One of the objects of our study was to determine which band is best for extracting the stand density and which windows size and NDVI are suitable for the corresponding band. We compared two QuickBird images acquired in different periods in the same area. A 2008 multispectral band and a 2013 panchromatic and a 2013 multispectral band were tested. A scatter diagram between the independent and dependent variables showed that the forest structure parameters NT and NSLMP display a generally linear relationship. Based on the correlation analysis results above, we selected the highest correlation coefficient as optimal combination of NDVI threshold and window size applying in the all unclassified forest stands, coniferous forest stands and broadleaf forest stands respectively.

The highest r value for unclassified stands was 0.79 (p < 0.01). For coniferous stand, the highest r value was 0.89 (p < 0.01) and for broad-leaf stands, the r value was 0.67 at p < 0.01. The results are shown in Table 1. Regression models between the NT and NSLMP derived from PAN band determined from QuickBird imagery were built using single variable regressions to predict forest parameters. The F-statistic indicated that the p-values were less than 0.001 and therefore, the models were highly significant. However, the non-linear regression model achieved the best results. We only present the non-linear regression model here and the model results are shown in Table 2. The prediction abilities of these models were evaluated by leave-one-out cross-validation scores (r^2^_cv_ were 0.66, 0.79, and 0.35 and the RMSEs were 13.97, 14.45, 10.97 corresponding with unclassified forest, coniferous forest and broadleaf forest respectively).

**Table 1:** Comparison result of stand density between two QuickBird images used in different forest from the Jiufeng area.

Year	Band	Unclassified	r	Coniferous	r	Broadleaf	r
**2013**	PAN	5×5、NDVI≥0.1	0.69***	3×3、NDVI≥0.3	0.88***	3×3、NDVI≥0.3	0.64***
	NIR	3×3、NDVI≥0.2	0.71***	3×3、NDVI≥0.4	0.89***	7×7、NDVI≥0.3	0.66***
**2008**	PAN	5×5、NDVI≥0.2	0.74***	3×3、NDVI≥0.3	0.86***	5×5、NDVI≥0.3	0.67***
	NIR	3×3、NDVI≥0.3	0.79***	3×3、NDVI≥0.3	0.86***	3×3、NDVI≥0.1	0.67***

Note

*** represents that the result of correlation analysis is significant at p = 0.001 level.

** represents that the result of fitting is significant at the p = 0.05 level.

**Table 2:** Non-linear regression model in different forest types based on NIR band.

Forest	NDVI	Window size	Coefficient of determination	P-value	F-statistic	RMSE	DOF
**Broadleaf**	≥0.1	3×3 window	0.4441	1.919e-05***	14.78	9.02	2 and 37 DF
**Coniferous**	≥0.3	3×3window	0.7933	1.422e-07***	38.38	12.60	2 and 20 DF
**Unclassified**	≥0.3	3×3window	0.7017	2.2e-16***	79.99	11.20	2 and 68 DF

Note

*** represents that the result of fitting is significant at p = 0.001 level. ** represents that the result of fitting is significant at the p = 0.05 level. DOF represents degree of freedom.

### Predicting stand density

**Fig 6:**
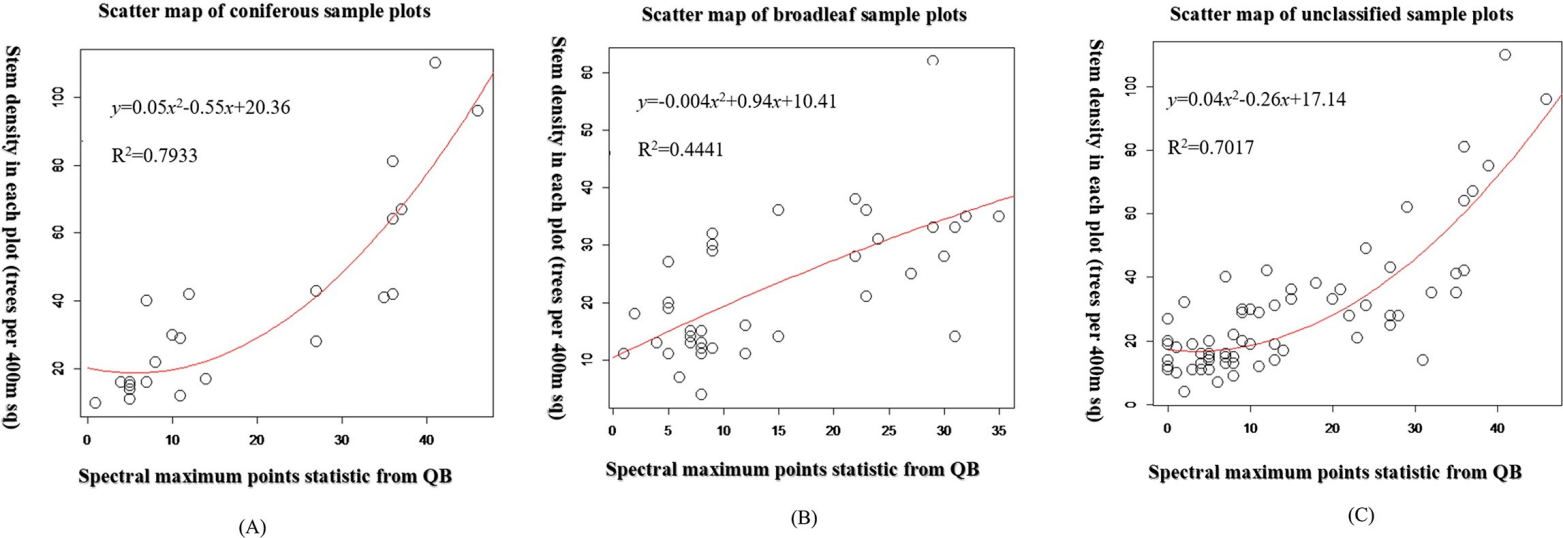
Number of trees / 400 square meters–observations of stand density against number of spectral local maximum points. Note: (A) Represents the scatter plot of stand density in coniferous sample plots. (B) Represents the scatter plot of stand density in broadleaf sample plots. (C) Represents the scatter plot of stand density in all stand sample plots.

**Fig 7:**
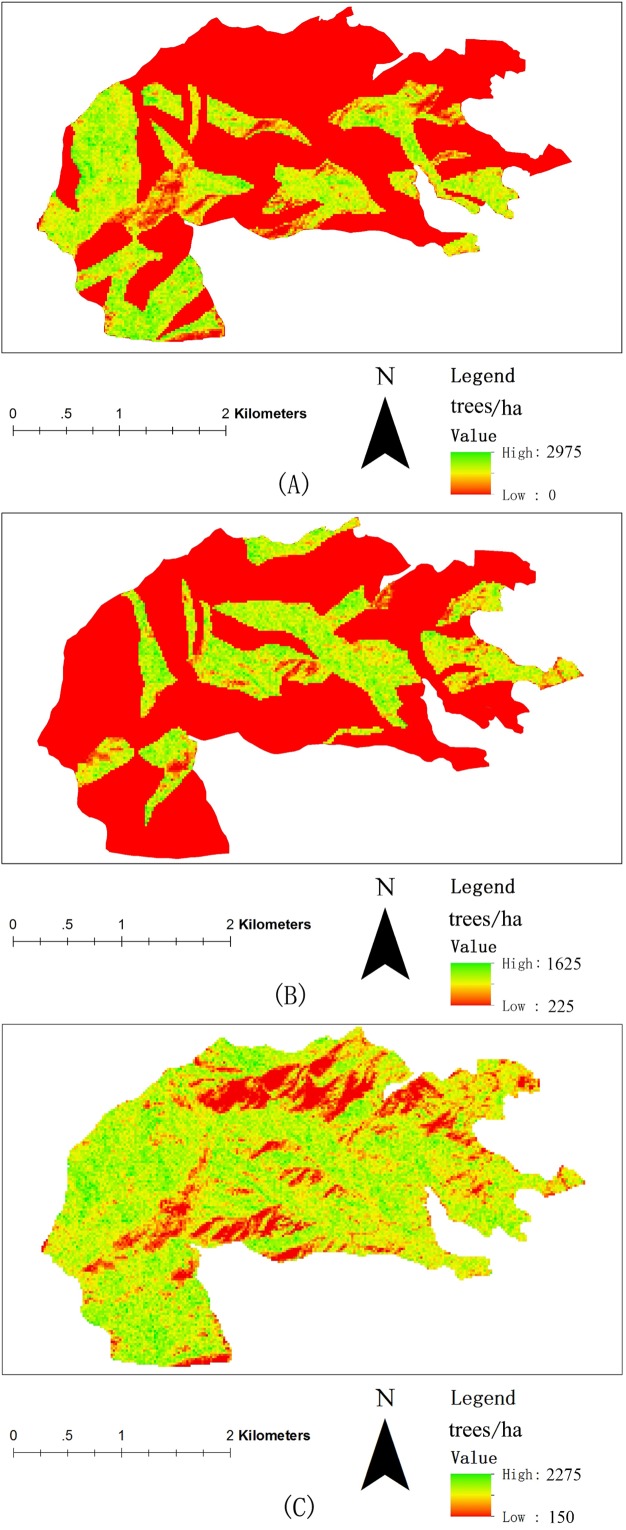
Number of trees / ha predicted by the non-linear regression model obtained by combining the forest inventory sample plot data and QuickBird imagery data using local maximum filtering of the study site in Jiufeng Park, Beijing. (A) Stand density of the coniferous stand. (B) Stand density of the broadleaf stand. (C) Stand density of the whole forest. Note: the 20 m × 20 m pixel size was used to produce these maps.

Fig 6 presents a scatter plot of the non-linear regression model. The Y-axis is the true (actual measured) tree density and the X-axis is tree density derived from images. As a result, the regression models can be considered predictive models for estimating the NT in the study area. Fig 7 illustrates stand density estimation results of the coniferous stands, broadleaf stands and unclassified stands in the study area.

## Discussion

We used a Pentax R-422N no-prism total station device in conjunction with DGPS to identify the positions of individual trees. Each tree was located precisely in the field, and the validity and feasibility of the survey method were demonstrated by its ability to locate each tree within two sample plots. Exact plot geolocation was a prerequisite for extracting forest parameters when compare field-measured stand density with stand density derived from imagery using remote sensing techniques. However, previous results were usually lack of verification, and those accuracies were still low in attempts to locate and classify species. The LM method used to extract NSLMP and estimate stand density, was improved by testing different window sizes and NDVI thresholds for different forest stand types. The proper window size selection is critical to obtain optimal results. We found that the window sizes are consistent (3 × 3) when applied in coniferous plot models, while window sizes varied when applied in broadleaf plot models. One reason could be that the mean crown size in broadleaf plots is larger than that in coniferous plots. The average crown size in coniferous plot was 2.68 m (North to South) and 2.95 m (East to West). The average crown size in broadleaf plots was 3.63 m (North to South) and 3.79 m (East to West). The standard deviation of coniferous crown size was 0.96 m. The stand deviation of broadleaf crown size was 1.52 m.

LM filtering is an effective and simple method for extracting NT. Wulder et al. applied LM filtering to the extraction of tree location and basal area. They also evaluated the precision of airborne and satellite high spatial resolution data and proposed error reduction methods for LM filtering of the high spatial resolution imagery used to locate trees [36]. Healthy conifers reflect near infrared energy strongly. The reflectance of NIR radiation is high from healthy vegetation with high exposure to sunlight. Moreover, because of differences in research content, sensor types, species and site conditions, research results are often inconsistent. Under normal conditions, estimations of coniferous forests dominated by pure stands of the same species usually produce satisfactory results, because of the single stand structure [37].

Our models can be used to estimate coniferous and broadleaf forest stand density because each model explains a significant proportion of the variables. Some studies have used different remote sensing data with different spectral and spatial resolution. These studies may have applied LM filtering to different study areas with different levels of forest density [14]. Our findings are consistent with those in previous studies. These include studies in which forest structure parameters, such as crown closure (CC) were transformed by principal transformation and predicted by textural methods using pan-sharpened SPOT imagery. This approach achieved an R^2^ of stepwise regression analysis of 0.79 [37]. Our results are also consistent with the results of Kayitakire et al., who used 1 m IKONOS-2 image texture information to model traditional forest parameters, including BA, height, circumference, density and age. They determined that the R^2^ of the best models ranged from 0.35 to 0.82 [38]. Remote sensing imagery with different spatial resolution may also lead to inconsistent results. Theoretically, each conifer canopy will have one single canopy spectral maximum because coniferous trees normally have fairly conical and symmetrical crowns. Therefore, the derived stand density should be more similar to the field-observed stand density in a coniferous forest sample plot. In addition, plots with low stand density generally have a higher predicted precision. The broadleaved tree canopy usually has a variable and irregular shape produces more than one spectral maximum point. This is why the correlation coefficients from coniferous forests are higher than those from broadleaf forests. In our study, the R^2^ in coniferous forest was 0.7415 which was higher than the 0.4422 value in broadleaf forest. Overall, our data affirms that coniferous forests provide more accurate results than broadleaf forests.

Several aspects are involved in improving the accuracy of stand density models. First, the appropriate window size for LM filtering is critical for generating crown reflectance maximum points. NDVI filtering is a key process that can be used to filter out non-crown points. Second, field data collection must be sufficiently precise and meticulous. Standing dead trees must be recognized because they will have a lower near-infrared reflectance than live trees and they would not be indicated as a tree peak in the spectral band. We obtained high accuracy because dead trees were identified and excluded during field data collection process. The position of the crown spectral maximum is likely to be different from the position of the tree stem, and even when trees in the sample plot were measured precisely using the total station. There were still omission and commission errors. The number of LM points was less than the actual stand density in most of the sample plots. Thus, we conclude that LM method is limited, especially when the stand density is high. That is true even when the NDVI is not used to filter the results.

We found that the results of LM filtering varied significantly for different tree species. The acquisition time of remote sensing imagery also significantly affected the LM results. For example, the leaves of most broadleaf trees fall in winter, which would produce a lower NDVI value for each tree. The results of extracting spectral maximum points by LM should be verified, and accuracy should increase if different tree species are considered. Results should be verified using high spatial resolution remotely sensed imagery with different acquisition times. Further work to refine LM techniques and more precisely extract individual tree crowns may be required, especially when applying the LM method in different locations.

The fusion of remote sensing data like Light Detection And Ranging (LiDAR) and the multispectral band could increase the accuracy of individual tree identification [14]. Fusion of remote sensing data should be the main research method used in future research. The results may be useful for forest stewards, ecologists, and silviculturists as a basis for more accurate modeling of forest structure and dynamics. Individual tree data would rarely be required after their amalgamation into stand parameters. However, computerized management systems may integrate the LM filtering method and models developed in this paper to achieve individual tree canopy identification and stand density predictions in similar research areas. This approach has significant potential and will be conductive to additional study.

## Conclusions

Coniferous forests provided the best results in the statistical models that were developed for coniferous forests, broadleaf forests and entire stands. This suggests that high accuracy estimations of coniferous tree density can be achieved by combining individual tree crown extraction from a high spatial resolution optical image and the ground measurements using both GPS and total station. The combination of a 3 × 3 window size and NDVI ≥ 0.3 threshold in coniferous forest produced the best result (coniferous forest R^2^ = 0.79, RMSE = 12.60). The best combination for broadleaf forest was a 3 × 3 window size and NDVI ≥ 0.1 with R^2^ = 0.44, RMSE = 9.02. The best combination of window size and NDVI threshold for an unclassified stand was a 3 x 3 window size and NDVI ≥ 0.3 with R^2^ = 0.70, RMSE = 11.20. The proposed methods use an advanced survey method that takes advantage of high spatial resolution imagery and optimal plot sampling strategies.

## Supporting information

S1 Data The underlying data. (RAR)
